# Success in increasing physical activity (PA) among patients with type 2 diabetes: a self-determination theory perspective

**DOI:** 10.1080/21642850.2018.1462707

**Published:** 2018-04-25

**Authors:** Anne M. Koponen, Nina Simonsen, Sakari Suominen

**Affiliations:** a Folkhälsan Research Center, and Department of Public Health, University of Helsinki, Finland; b School of Health and Education, University of Skövde, Skövde, Sweden; c Department of Public Health, University of Turku, Finland

**Keywords:** Diabetes mellitus, sedentary lifestyle, adherence, physical activity, self-determination theory

## Abstract

**Background:**

Increased physical activity (PA) is crucial for achieving and maintaining glycemic control and is beneficial for overall well-being of patients with type 2 diabetes as well. Despite that, many patients fail to make changes in their exercise behavior. Self-determination theory (SDT) addresses this problem and suggests that perceived autonomy support, autonomous motivation and self-care competence play a key role in the process of health behavior change. This study investigated the impact of these three factors on success in increasing PA among patients with type 2 diabetes but considered also the role of other important life-context factors, such as mental health, stress and social support. The effect of these other factors may outweigh the effect of SDT constructs; however, previous studies based on SDT have largely overlooked them.

**Methods:**

This cross-sectional mail survey was carried out in 2011. Out of 2866 respondents, those who had been over 2 years in care in their present and principal primary care health center and had during the past two years tried to increase PA either with or without success (*n* = 1256, mean age 63 years, 52% men), were included in this study. Logistic regression and mediation analyses were the main methods used in the data analysis.

**Results:**

Autonomous motivation predicted success in increasing PA even after controlling for the effect of other important life-context factors. Other predictors of success were felt energy, good perceived health, younger age and less social support. Autonomous motivation mediated the effect of perceived autonomy support from a doctor on success in increasing PA.

**Conclusion:**

The results were in line with SDT showing the importance of autonomous motivation for success in increasing PA. Doctor–patient relationships and lifestyle interventions should focus on promoting self-motivated reasons for health behavior change.

## Introduction

Diabetes is a growing burden all over the world. It is estimated that 425 million adult people had diabetes in 2017 and this number is expected to rise to 629 million by 2045 (International Diabetes Federation, 2017). Diabetes is a major public health concern also in Finland. Approximately 550,000 have diabetes of which 500,000 is of type 2 (National Institute for Health and Welfare [THL], 2016). Thus, about 10% of the total population has type 2 diabetes. Aging of the population, sedentary lifestyle, increased consumption of high-energy dense food and obesity are the main causes for this increasing epidemic. Ideal care for diabetes, in order to reach glycemic control and avoid diabetes related complications, includes healthy diet, regular physical exercise and medication when needed (American Diabetes Association, 2017).

Adherence to care recommendations may mean a great change in daily routines. Diet should be changed and increased PA added to own and maybe to the family schedule, as well. Such great changes may be found uninteresting and have been shown to be difficult to accomplish (Madden, Loeb, & Smith, 2008). Patients should internalize the value of behavior change, that is, assimilate the behavior into the self and take responsibility for its regulation (Teixeira, Silva, Mata, Palmeira, & Markland, 2012). Only then, any long-term consistency in behavior change, such as increase in PA, is regarded to be possible (Ryan, Patrick, Deci, & Williams, 2008).

Self-determination theory (SDT) is a general theory of human motivation that has been increasingly used to explain the internalization process leading to autonomous motivation for permanent behavior change (Fortier, Sweet, et al., 2012; Ng et al., 2012; Teixeira, Carraça, Markland, Silva, & Ryan, 2012). According to SDT, people can be placed on a motivational continuum ranging from autonomous (self-determined) motivation to more controlling in nature (Ryan & Deci, 2000). Motivation is seen as psychological energy directed at a particular goal, the energy that moves people to act (Ryan, Lynch, Vansteenkiste, & Deci, 2011). The importance of motivational quality in addition to its quantity is emphasized (Patrick & Williams, 2012).

In the most autonomous forms of motivation, exercise is performed because (a) it gives enjoyment and satisfaction (intrinsic motivation), (b) a person has integrated exercise as a central value in his/her value system (integrated regulation) or (c) a person just values exercise as important for health (identified regulation). When the motivation is more controlled, a person exercises in order (a) to get approval or praise or, alternatively, to avoid disapproval or feelings of guilt (introjected regulation) or (b) to get an external reward, avoid a punishment or to comply with social pressures (external regulation). Amotivated persons have no intention to exercise. The more autonomous the regulation is, the more likely a long-term increase of PA will be achieved (Ryan et al., 2008; Wilson & Rodgers, 2004).

The individual position on the motivational continuum is partly determined by personality differences regarding autonomy and life aspirations (intrinsic vs. extrinsic), (Ryan et al., 2008). Individuals with intrinsic aspirations value goals that are internal to the self, such as personal growth and physical health. Thus, they evidence higher levels of autonomy, and intrinsic aspirations have been shown to be associated with better health behavior and health. Instead, those with extrinsic aspirations value external goals such as wealth, fame and physical attractiveness and show lower levels of autonomy and greater health risk behaviors and poorer health (Deci & Ryan, 2000; Patrick & Williams, 2012; Ryan et al., 2008).

Differences in personality explain only a part of the motivational quality. Autonomy supportive environments, such as an autonomy supportive health care climate, play a great role in enhancing autonomous motivations (Ryan & Deci, 2000; Ryan et al., 2008). People are seen to have innate and universal psychological needs for autonomy (feeling self-determined), competence (feeling effective) and relatedness (feeling understood and cared for). Feelings of being the initiator of the behavior, competent and connected to others, have intrinsic value and are essential for well-being and behavioral persistence. Environments that satisfy these basic needs may increase a person’s autonomous regulation of a specific behavior (Ryan et al., 2008). Behavior change interventions and doctor–patient interactions should focus primarily on promoting self-motivated reasons to change and not rely on external support or incentives. The primary aim should be integration of change within personality rather than behavior change per se. That is, the value of behavior change should be internalized and integrated with one’s sense of self, values and goals. Otherwise, behavior change is unlikely to be permanent (Teixeira, Silva, et al., 2012).

Thus, an autonomy supportive health care climate is the one that satisfies patients’ needs for relatedness, autonomy and competence. People feel relatedness with health care personnel when they are respected and understood, and care is available when needed. An emphatic care environment increases the likelihood that patients listen to suggestions for better health behaviors and adopt them. Feelings of autonomy or self-determination are enhanced by providing meaningful rationales for change, by avoiding pressure and blame, and by listening to patients’ opinions and considering different options with them e.g. regarding suitable exercise. Feelings of competence can be enhanced by setting optimal goals together with the patient and by giving practical advice and supportive feedback (Ryan et al., 2008). According to the SDT model of health behavior change, an autonomy supportive health care climate affects autonomous motivation and perceived competence, which in turn predict health behaviors and health outcomes (Ng et al., 2012).

Increasing research evidence shows a positive association between autonomous motivation and short and long-term PA, as well as between autonomy supportive health care climate and autonomous motivation for self-care (Fortier, Duda, Guerin, & Teixeira, 2012; Koponen, Simonsen, & Suominen, 2017a, 2017b; Ng et al., 2012; Teixeira, Carraça, et al., 2012). Castonguay and Miquelon (2017) found that only participants who had a self-determined profile observed the recommended PA guidelines, that is, they practiced at least 150 minutes of moderate-to-vigorous PA per week for 20–30 minutes per session.

However, besides autonomous motivation, many other personality related factors, as well as factors in the larger life context, may affect adherence to care recommendations and should be taken into account when studying patients with type 2 diabetes. The effect of these other factors has not been systematically analyzed in previous studies based on SDT (Koponen, Simonsen, & Suominen, 2015). Depressive symptoms, which are more common among patients with diabetes than in the general population (Ali, Stone, Peters, Davies, & Khunti, 2006; Anderson, Freedland, Clouse, & Lustman, 2001; Nouwen et al., 2010), have been found to be associated with poor self-management of diabetes (Ali et al., 2006; De Groot, Anderson, Freedland, Clouse, & Lustman, 2001; Dirmaier et al., 2010; Egede & Ellis, 2010; Gonzalez et al., 2007). Also, stressful life experiences, including stress caused by the chronic illness (Gonzalez, Fisher, & Polonsky, 2011), may hinder success in increasing PA. On the other hand, a strong sense of coherence (Antonovsky, 1987) and social support from significant others (Williams, Freedman, & Deci, 1998) may help to make changes in health behaviors. Our earlier studies showed that besides autonomous motivation also felt energy, using oral medication only as diabetes medication and younger age were positively associated with engagement in physical activity and success in weight management (Koponen et al., 2017a; Koponen,‌Simonsen,‌&‌Suominen. 2017c).

This study investigated (1) the impact of perceived autonomy support from one’s physician, autonomous motivation and self-care competence on success in increasing PA among patients with type 2 diabetes, (2) the role of the other important life-context factors (perceived health, medication, duration of diabetes, mental health, stress and social support) for success in increasing PA and (3) the possible mediating role of autonomous motivation and self-care competence between perceived autonomy support and success in increasing PA.

The hypotheses of the study were that (1) perceived autonomy support from one’s physician, autonomous motivation and self-care competence predict success in increasing PA even after the effect of the other important life-context factors has been controlled for, and (2) the effect of perceived autonomy support from one’s physician on success in increasing PA is mediated by autonomous motivation and self-care competence.

## Materials and methods

### Cross-sectional survey

The study was carried out as a mail survey in 2011. Patients with type 2 diabetes were identified from the register of the Social Insurance Institution of Finland (Kela). Kela is a Finnish government agency (funded directly from taxation) in charge of settling benefits under national social security programs. Kela keeps the register of persons entitled to a special reimbursement for medicines for chronic diseases such as diabetes. The sample of the present study was collected among persons who fulfilled the following inclusion criteria:

- had entitlement to a special reimbursement for medicines used in the treatment of type 2 diabetes (ICD-10 code, E11) in 2000–2010, and the right was valid in September 2011 and onward,
- born in 1936–1991 (20–75 years), alive and had no safety prohibition at the time of the data collection,
- Finnish as a native language,
- one of the five study municipalities as a place of residence.

Based on power-analysis, a sample of 5167 persons out of a total of 7575, who fulfilled the inclusion criteria, was collected: 2000 persons from the two large municipalities and all persons from the three small municipalities. There were 2962 (57%) men and 2205 women (43%) in the sample, corresponding to gender rates in the total population of patients with type 2 diabetes in the study municipalities.

A pilot study (*n* = 50) was carried out in May 2011 by the authors of this study in order to test the first version of the research questionnaire. Some revisions were made, and the final version was mailed to respondents by Kela in September 2011. Non-respondents got two reminders: the first one was sent out in October, and the second one with a new copy of the questionnaire in November. The final response rate was 56% (range 54–59% across municipalities, *n* = 2866). Sex and age had effect on the response rate: women responded slightly more often (57%) than men (54%), and the response rate was highest (63%) in the oldest age group (65–75 years), lower (55%) in the age group of 55–64 years and lowest (36%) in the age group of 20–54 years.

### Participants

Eighty-four percent of the respondents had been over two years in diabetes care in their current and principal primary care health center. About a third of them (31%, *n* = 686) had during the two last years tried and succeeded to increase PA, and about a quarter (26%, *n* = 570) had tried without success. Twenty-nine percent (*n* = 630) reported having no need to increase PA, 9% (*n* = 191) intended to do so in the near future, and 6% (*n* = 133) had no intention for change. Ninety-seven cases were missing. Only those respondents, a total of 1256 patients, who had tried to increase PA during the last two years either with success (successors) or without success (non-successors) were included in the present study. (Table 1.)

**Table 1.** Sociodemographic background factors of respondents.

	*N* In care over 2 years *N* = 2307	%	*N* In care over 2 years and has tried to increase PA during the last 2 years *N* = 1256	%
Sex Man Woman Total (Missing)	1274 1027 2301 (6)	55.4 44.6 100	645 608 1253 (3)	51.5 48.5 100
Age 27–54 years 55–64 years 65–75 years Total (Missing)	268 845 1152 2265 (42)	11.8 37.3 50.9 100	180 498 552 1230 (26)	14.6 40.5 44.9 100
Marital status Single Married Cohabiting Divorced Widowed Total (Missing)	220 1383 156 323 204 2286 (21)	9.6 60.5 6.8 14.1 8.9 100	129 719 98 191 107 1244 (12)	10.4 57.8 7.9 15.4 8.6 100
Professional education Upper secondary education (vocational school or less) Higher education (college, polytechnic, university) Total (Missing)	1350 906 2256 (51)	59.8 40.2 100	731 504 1235 (21)	59.2 40.8 100
Principal activity Working Retired because of old age Retired because of chronic illness Other Total (Missing)	552 1283 298 138 2271 (36)	24.3 56.5 13.1 6.1 100	340 653 175 66 1234 (22)	27.6 52.9 14.2 5.3 100
Diabetes medication Oral Insulin Tablets+insulin Other Total (Missing)	1660 119 424 44 2247 (60)	73.9 5.3 18.9 2.0 100	894 56 253 23 1226 (30)	72.9 4.6 20.6 1.9 100
Service provider Municipal Private Total (Missing)	1856 364 2220 (87)	83.6 16.4 100	990 224 1214 (42)	81.5 18.5 100
Family or regular doctor No Yes Total (Missing)	558 1694 2252 (55)	24.8 75.2 100	303 923 1226 (30)	24.7 75.3 100
Body Mass Index Underweight <18.5 Normal weight 18.5–24.9 Overweight 25.0–29.9 Class I obesity 30.0–34.9 (moderately obese) Class II obesity 35.0–39.9 (severely obese) Class III obesity ≥40.0 (very severely obese) Total (Missing)	6 311 818 642 300 165 2242 (65)	0.3 13.9 36.5 28.6 13.4 7.4 100	2 101 413 415 185 111 1227 (29)	0.2 8.2 33.7 33.8 15.1 9.0 100
Success in increasing physical activity No need for change Has changed behavior Has tried to change but has not succeeded Not yet but intends to change in the near future Has not and has no intention to change in the near future Total (Missing)	630 686 570 191 133 2210 (97)	28.5 31.0 25.8 8.6 6.0 100	686 570 1256	54.6 45.4 100

The mean age of the respondents was 63 years (standard deviation (SD) 8 years, range 31–75 years), 52% of them were men, and 92% were overweight or obese. Over half (53%) of the respondents were retired because of old age, 58% were married, and 41% had a higher professional education. The majority (82%) of the respondents had a municipal primary care health center as their primary care setting in diabetes care, 75% had a family or regular doctor, and 73% used oral medication only for diabetes therapy. These rates are quite comparable with all respondents who had been in care in their principal primary care health center for longer than two years and with the entire sample, except for gender, age and BMI: those who had tried to increase PA were more often female, younger and obese as compared to the rest of the participants (Koponen et al., 2015) (Table 1).

### Ethical issues

The research plan was accepted by the Ethical Committee of the Hjelt Institute, University of Helsinki, and the research permission was received from Kela. A qualified statistician who worked at Kela collected the sample, and the questionnaires were posted from Kela. Respondents filled the questionnaires and sent them directly to the researchers by mail. Questionnaires were provided by an identification number in order to check for non-response. The researchers had no possibility to identify the identity of respondents, and only the researchers saw the responses.

### Variables

Descriptions of the measures used in this study are presented in Table 2. Cronbach’s alphas of the measures chosen for the final analyses varied from 0.75 to 0.95 and can be regarded acceptable (higher than 0.70) or excellent (higher than 0.80), (Andresen, 2000).

**Table 2.** Measures used in the study

SDT variables	
Perceived autonomy support (from one’s physician)	The short 6-item form of health care climate questionnaire (HCCQ, n.d.), (range 1 = fully disagree, 5 = fully agree, Cronbach’s alpha reliability *α *= 0.95). Example item: I feel that my physician has provided me choices and options. (http://www.selfdeterminationtheory.org/)
Autonomous motivation	Autonomous regulation (motivation) scale B. Five items from the treatment self-regulation questionnaire (TSRQ, n.d.), (range 1 = not at all true, 7 = very true, *α *= 0.83). Example item: The reason I follow my diet and exercise regularly is that I personally believe that these are important in remaining healthy. (http://www.selfdeterminationtheory.org/)
Self-care competence	The 4-item perceived competence for diabetes scale (PCS, n.d.), (range 1 = fully disagree, 5 = fully agree, *α *= 0.93). Example item: I feel confident in my ability to manage my diabetes. (http://www.selfdeterminationtheory.org/)
*Mental health dimensions*	
Energy	The 4-item scale measuring energy during the last four weeks from the RAND-36-Item Survey, 1.0 (range 0–100%, *α *= 0.85). Example item: How much of the time during the past 4 weeks did you have a lot of energy? (Hays, Sherbourne, & Mazel, 1993.)
Emotional well-being	The 5-item RAND-36 scale measuring emotional well-being during the last four weeks (range 0–100%, *α *= 0.84). Example item: How much of the time during the past 4 weeks have you felt so down in the dumps that nothing could cheer you up? (Hays et al., 1993.)
Sense of coherence	The short 13-item scale (range 1 = weak, 7 = strong, *α *= .80, five items reversed). Example item: Do you have feeling that you don’t really care about what goes on around you? (1 = very often, 7 = very seldom or never), (Antonovsky, 1987.)
Depression	Diagnosed depression (1 = no, 2 = yes).
*Experienced stress and social support*	
Life stress	Experienced stress during the last year (12 months) in the 10 life areas e.g. own health and economic situation (range 1 = not at all, 4 = very much). Based on the Living with Diabetes Study. School of Population Health. University of Queensland. (Donald et al., 2012).
Social support in diabetes	A 12-item scale measuring support and help received from friends, relatives and health care personnel (range 1 = fully disagree, 5 = fully agree, *α *= .75). Example item: When I feel bored, depressed or desperate, my friends and family are ready to listen to me. (Toljamo, 1999). The scale is based on social support scales by Brandt and Weinert (1981), Goodenow, Reisine, and Grady (1990), Norbeck, Lindsey, and Carrieri (1981; 1983), Stewart and Tilden (1995) and Weinert (1987).
*BMI and physical health*	
Body mass index (BMI)	Counted based on answers to two questions: About how tall are you?, About how much do you weigh with light clothes? BMI* *= ((P2/(P1*P1))*10000.
Perceived health	A single-item scale, range 1 = excellent, 2 = very good, 3 = good, 4 = quite poor, 5 = poor. The scale was dichotomized: 1 = good (1–3), 2 = poor (4–5).
*Physical activity and advice*	
Success in increasing PA	Have you changed your health behavior during the last two years (24 months) in order to increase physical activity? 1 = I have tried but failed, 2 = I have changed my health behavior http://www.palmenia.helsinki.fi/ikihyva/Ikihyva_perusraportti_2008_70.pdf
Intensity of physical activity	How often do you exercise physically in your spare time for at least 30 minutes to the extent that you at least slightly lose your breath and perspire?, range 1 = I cannot perform exercise due to illness or handicap, 7 = daily. (HBHAF-questionnaire) http://urn.fi/URN:ISBN:978-952-245-931-2
Exercise counseling	Have you gotten in your current and principal primary care health center information, advice and guiding on suitable physical exercise? (range 1 = not at all, 2 = too little, 3 = enough).

Averaged sum scales for perceived autonomy support from one’s physician, autonomous motivation, self-care competence, energy, emotional well-being, a sense of coherence, life stress and social support in diabetes were calculated. The respondent was included in the analysis if she/he had answered at least to 70% of the scale items (Table 2).

Body mass index (BMI) was calculated by dividing weight in kilograms by the square of height in meters. Participants were classified as underweight if their BMI was under 18.5, normal weight if BMI ranged from 18.5 through 24.9, and overweight if their BMI ranged from 25 through 29.9. We divided obesity (BMI ≥ 30) into 3 levels: BMI of 30 through 34.9, class 1, moderately obese; BMI of 35 through 39.9, class 2, severely obese; and BMI of 40 or higher, class 3, very severely obese (Mokdad et al., 2003) (Tables 1–2).

## Statistical methods

The data were first analyzed by descriptive analysis methods. The baseline associations between independent variables, covariates and dependent variables were tested with Pearson chi²-tests, *t*-tests or one-way analysis of variance depending on the measurement scale of the variable of interest. Before the final logistic regression analyses, correlations between the study variables were explored by Pearson or Spearman correlations (when one or both variables were dichotomous, ordinal scale). The level of statistical significance was set at *p* < .05. Of the independent variables that measured the same phenomena, such as different dimensions of mental health (energy, emotional well-being, diagnosed depression and a sense of coherence), only the one that correlated most strongly with success in increasing PA was chosen to the final logistic regression analyses in order to avoid multicollinearity problems.

In the mediation analysis between perceived autonomy support, autonomous motivation, self-care competence and success in increasing PA, the instructions reported by Baron and Kenny (1986) were followed. First, the mediator was regressed on the independent variable. Second, the dependent variable was regressed on the independent variable. Third, the dependent variable was regressed on both the independent variable and on the mediator. A mediation exists if the predicted associations hold on each step of the analysis and if the effect of the independent variable on the dependent variable is less in the third step than in the second step. The mediation is perfect if the independent variable has no effect when the mediator is controlled. Statistical significance of the mediation was calculated by the Sobel test (Preacher & Leonardelli, n.d.; Preacher & Hayes, 2004). Statistical analyses were performed using SPSS version 23. List-wise deletion of missing data was used.

### Ethics statement

The research plan was accepted by the Ethical Committee of the Hjelt Institute, University of Helsinki, and the permission to conduct the study was received from Kela. The respondents gave their consent to participate by the act of returning the questionnaire.

## Results

### Preliminary analyses

Both the successors and non-successors had been equally advised to exercise regularly (94%/92%, respectively, *p* > .05) but the successors reported more frequently (56%/48%, *p* < .01) that they had got enough information, advice and guidance regarding suitable exercise for them. About a third (34%) of the successors and 10% of the non-successors used to exercise at least 4 times a week at least 30 minutes on each occasion to the extent that they at least slightly lost their breath and perspired (*p* < .001). About a half (53%) of the successors and 62% of the non-successors were obese (*p* < .001).

The four variables measuring different dimensions of mental health (energy, emotional well-being, diagnosed depression, a sense of coherence) correlated moderately or strongly with each other (−0.40 – 0.79), (Taylor, 1990). Only the correlation between a sense of coherence and depression was weaker (−0.34). Correlations between the four variables and success in increasing PA were weak (≤ 0.24). Of these four variables, energy correlated most strongly with success in increasing PA (0.24, *p* < .001), whereas Spearman correlations between a sense of coherence, emotional well-being and diagnosed depression, and success in increasing PA were 0.12 (*p* < .001), 0.16 (*p* < .001) and −0.01 (*p* > .05), respectively. Therefore, energy was included as an independent variable to the multivariate logistic regression analyses.

### Primary analyses

Table 3 shows that autonomous motivation, self-care competence and perceived autonomy support correlated positively with success in increasing PA but the correlations were quite weak (0.29, 0.18 and 0.08, respectively). In addition, energy and social support correlated slightly positively (0.24, 0.09) and poor health, stress and insulin medication slightly negatively with success in increasing PA (−0.19, −0.12 and −0.06, respectively).

**Table 3.** Pearson/Spearman correlations^a^ between the study variables (*n* = 1256)

	1	2	3	4	5	6	7	8	9	10	11	12
1. Perceived autonomy support												
2. Autonomous motivation	.26***											
3. Self-care competence	.36***	.42***										
4. Sex (1 = man, 2 = woman)	−.08**	.11***	−.02									
5. Age	.03	.12***	.12 ***	.02								
6. Education (1 = less than higher education 2 = higher education)	−.02	−.03	−.07*	.03	−.11***							
7. Duration of diabetes	.01	.01	−.01	−.04	.19 ***	−.04						
8. Diabetes medication (1 = tablets only, 2 = other)	−.02	.02	−.01	−.03	−.12 ***	−.01	.26***					
9. Perceived health (1 = good, 2 = poor)	−.21***	−.16***	−.28 ***	.02	.05	−.07 *	.13***	.11***				
10. Energy	.25***	.23***	.38 ***	−.09**	.11***	.00	−.05	−.11***	−.46***			
11. Stress	−.19***	−.11***	−.28 ***	.24 ***	−.33 ***	.12 ***	.00	.08*	.22***	−.48***		
12. Social support	.42***	.36***	.33 ***	.03	.07**	−.06 *	−.05*	−.01	−.21***	.38***	−.30***	
13. Increased PA (1 = not succeeded, 2 = succeeded)	.08**	.29***	.18 ***	.02	−.01	.04	−.01	−.06*	−.19***	.24***	−.12***	.09**

^a^Spearman correlation was used when one or both variables were dichotomous (ordinal scale).

*p < .05.

**p < .01.

***p < .001.

Table 4 shows that autonomous motivation was associated with success in increasing PA even after the effect of other important life-context factors was controlled for, but the other SDT variables (perceived autonomy support and self-care competence) were not. Also, energy was positively, and higher age, poor health and social support negatively associated with success in increasing PA.

**Table 4.** Multivariate logistic regression models on the associations of perceived autonomy support (from one’s physician), autonomous motivation, self-care competence and important confounding factors with success in increasing physical activity

	Model 1 (95% CI)	Model 2 (95% CI)	Model 3 (95% CI)	Model 4 (95% CI)
Perceived autonomy support Autonomous motivation Self-care competence	0.98 (0.87–1.09) 1.64*** (1.46–1.85) 1.10 (0.93–1.29)	0.96 (0.85–1.08) 1.68*** (1.49–1.90) 1.13 (0.96–1.34)	0.91 (0.80–1.03) 1.73*** (1.52–1.96) 1.07 (0.90–1.28)	0.98 (0.86–1.13) 1.78*** (1.54–2.05) 0.98 (0.81–1.20)
Sex (1 = man, 2 = woman) Age Professional education (1 = less than higher education, 2 = higher education)		0.94 (0.73–1.20) 0.98 (0.97–1.00) 1.31* (1.02–1.68)	0.93 (0.71–1.21) 0.98 (0.97–1.00) 1.30 (1.00–1.69)	1.03 (0.77–1.37) 0.97** (0.95–0.99) 1.22 (0.92–1.61)
Duration of diabetes Medication (1 = tablets only, 2 = other) Perceived health (1 = good, 2 = poor)			1.02 (0.99–1.05) 0.76 (0.56–1.02) 0.50*** (0.38–0.66)	1.03 (1.00–1.05) 0.75 (0.55–1.04) 0.67** (0.49–0.91)
Energy Stress Social support				1.02*** (1.01–1.02) 0.85 (0.60–1.20) 0.71* (0.54–0.93)
Nagelkerke *R* Square Percentage of correct predictions *N*	0.11 63.4 1177	0.12 63.2 1133	0.17 63.7 1071	0.19 65.0 965

*p < .05.

** *p* < .01.

*** *p* < .001.

**Figure 1.**
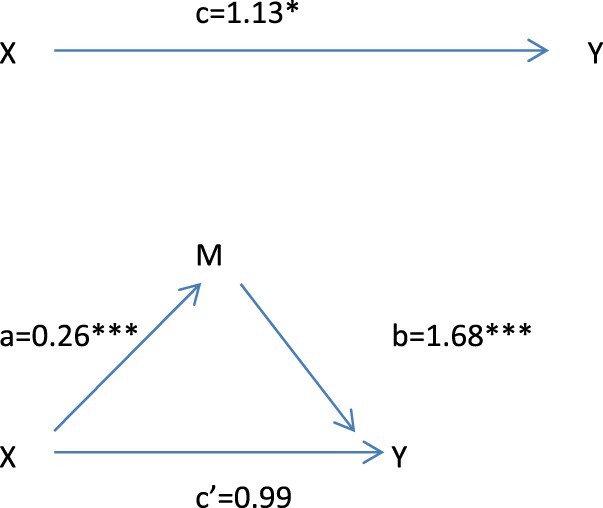
Mediation effect between perceived autonomy support, autonomous motivation and success in increasing physical activity. Note: X = Perceived autonomy support; Y = Success in increasing PA; M = Autonomous motivation; a = Effect of X on M; b = Effect of M on Y when X is controlled for; c = Effect of X on Y; c’ = Effect of X on Y when M is controlled for.

Table 5 shows that the association between autonomous motivation and success in increasing PA did not diminish after the effect of self-care competence was controlled for. Thus, self-care competence did not mediate the effect of autonomous motivation on success in increasing PA. Perceived autonomy support was associated with autonomous motivation. Perceived autonomy support was associated also with success in increasing PA but this association disappeared after the effect of autonomous motivation was controlled for which indicates perfect mediation: perceived autonomy support was associated with increased PA through autonomous motivation. (Table 5, Figure 1)

**Table 5.** Mediation analysis between perceived autonomy support (from one’s physician), autonomous motivation, self-care competence and success in increasing physical activity (PA), linear/logistic regression models.

	Beta	OR (95% CI)	*N*
1. Autonomous motivation x self-care competence	0.42***		1215
2. Autonomous motivation x success in increasing PA		1.67 *** (1.51–1.86)	1231
3. Autonomous motivation x success in increasing PA Self-care competence x success in increasing PA		1.63*** (1.46–1.83) 1.10 (0.95–1.28)	1215
Sobel test: *z* = 1.27, SE = 0.02, *p* = .20			
1. Perceived autonomy support x autonomous motivation	0.26***		1191
2. Perceived autonomy support x success in increasing PA		1.13* (1.03–1.25)	1207
3. Perceived autonomy support x success in increasing PA Autonomous motivation x success in increasing PA		**0.99** (0.89–1.11) 1.68*** (1.50–1.87)	1191
Sobel test: *z* = 6.53, SE = 0.02, *p* = .000			

Notes: The bold value indicates mediation which exists if the predicted associations hold on each step of the analysis and if the effect of the independent variable on the dependent variable is less in the third step than in the second step. 1 = the mediator regressed on the independent variable; 2 = the dependent variable regressed on the independent variable; 3 = the dependent variable regressed on both the independent variable and on the mediator.

*p < .05.

** *p* < .01.

*** *p* < .001.

## Discussion

This study aimed to identify factors that predict success in increasing PA among patients with type 2 diabetes. Based on SDT, the focus was on evaluating the role of perceived autonomy support from one’s physician, autonomous motivation and self-care competence but also the effect of other important life-context factors, such as stress, social support and mental health, was investigated. The previous research based on SDT has largely overlooked the effect of these other factors even though they may play even a greater role in health behavior change than the three SDT variables. For example, the patient may be autonomously motivated to increase PA but because of depression, poor health or stressful life-situation behavior change is difficult to accomplish.

The respondents had been over two years in care in their current and principal primary care health center, and had during the past two years tried to increase PA either with or without success. We assumed that care received over two years of time had affected their success in increasing PA.

Almost all respondents had been advised to exercise regularly but the successors reported more often that they had got information, advice and guidance regarding suitable exercise for them, and they also exercised more and were more seldom obese compared with the non-successors. Success in increasing PA was predicted by strong autonomous motivation, energy, good perceived health, less social support and young age. An autonomy supportive health care climate was not directly associated with success in increasing PA but through autonomous motivation.

Our results are in line with SDT and the previous studies, which have stressed the importance of autonomous motivation for short and long-term physical activity (Ryan et al., 2008; Teixeira, Carraça, et al., 2012). This study also confirms the results of our previous study (Koponen et al., 2017a), which investigated engagement in physical activity in the whole data, including also those with no reported need or intention to increase PA. The previous study showed that of all measured explanatory factors, autonomous motivation was most strongly associated with engagement in PA, and autonomous motivation mediated the effect of perceived autonomy support on patients’ PA.

It seems, as suggested by Patrick and Williams (2012), that the more autonomously motivated the person is toward a certain behavior, the more effort he/she is ready to invest on that behavior. Autonomous motivation was a better predictor of success in increasing PA than self-care competence. Moreover, self-care competence did not mediate the effect of autonomous motivation on success in increasing PA as could be predicted by the model by Williams et al. (1998). The results suggest that health care practitioners are able to help patients’ success in increasing PA by promoting their autonomous motivation for change.

The effect of energy on success in increasing PA was stronger than the effect of diagnosed depression, which in many other studies has been found to be associated with care adherence (Egede & Ellis, 2010; Gonzalez et al., 2007; 2008). Our earlier studies similarly showed that energy was a better predictor of engagement in PA (Koponen et al., 2017a) and success in weight management (Koponen et al., 2017c) than diagnosed depression. These results support the notion of Gonzalez et al. (2007) that continuous depressive symptom scores are better predictors of non-adherence to exercise than categorically defined major depression.

Social support was negatively associated with success in increasing PA. This is consistent with findings in our previous studies, which showed that social support was negatively associated with engagement in PA (Koponen et al., 2017a) and success in weight management (Koponen et al., 2017c). These results are somewhat surprising but may be explained by the fact that those who need more support in diabetes care have poorer health and thus may have compromised the ability to follow care recommendations.

### Strengths and limitations of the study

The strengths of our study were the high response rate, large sample size and a large number of variables measuring important life-context factors. Thus, we were able to investigate the relative effect of the central SDT constructs on success in increasing PA compared with other important explanatory factors. A limitation of this study was the cross-sectional nature of the study, which makes it difficult to confirm the directionality of the hypothesized relations. In order to diminish this problem, we analyzed only those respondents who had been for a longer time than two years in care in their current and primary care health center and who had during the past two years tried to increase PA either with or without success. A majority (75%) of the respondents had a family doctor or a ‘regular’ doctor. Therefore, we believe that care and autonomy support received from one’s doctor had influenced the patient’s motivation for self-care, and ultimately success in increasing PA. All respondents were Finnish speaking and almost all native Finns, which may diminish the generalizability of the results to cultures with different perceptions of autonomy.

In summary, the results of this study supported SDT by showing that even after controlling for the effect of many central life-context factors autonomous motivation remained the strongest predictor of success in increasing PA. The study also showed, consistently with the results from previous studies, that only a minority of patients with type 2 diabetes performed PA according to recommendations (Koponen et al., 2017a; Finnish Diabetes Association). Therefore, it is extremely important to understand predictors of health behavior change and maintenance of change. SDT-based lifestyle interventions aiming to promote autonomous motivation for change could be tested more widely in primary health care.

## Conclusions

Findings of this study supported SDT by showing that autonomous motivation was the strongest predictor of success in increasing PA. Doctor–patient interactions and lifestyle interventions should focus on promoting self-motivated reasons to change and integration of change within personality.
